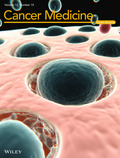# Cover Image

**DOI:** 10.1002/cam4.6501

**Published:** 2023-08-31

**Authors:** Adrian Fairey, Robert J. Paproski, Desmond Pink, Deborah L. Sosnowski, Catalina Vasquez, Bryan Donnelly, Eric Hyndman, Armen Aprikian, Adam Kinnaird, Perrin H. Beatty, John D. Lewis

## Abstract

The cover image is based on the Research Article *Clinical Analysis of EV‐Fingerprint to Predict Grade Group 3 and Above Prostate Cancer and Avoid Prostate Biopsy* by Adrian Fairey et al., https://doi.org/10.1002/cam4.6216.